# Recombinant Fibrinogen‐Gamma‐Chain as a Crosslinker of Thiol‐ene Hydrogels

**DOI:** 10.1002/bip.70097

**Published:** 2026-04-03

**Authors:** Domenic Schlauch, Charlotte Selin Güler, Marina Harzi, Selin Kara, Antonina Lavrentieva, Iliyana Pepelanova

**Affiliations:** ^1^ Leibniz University Hannover Hanover Germany

**Keywords:** fibrinogen‐gamma chain, gelatin norbornene, GelNB, recombinant hydrogel, thiol‐ene reaction

## Abstract

Hydrogels based on thiol‐ene step‐growth chemistry have gained increased attention due to their superior properties over the currently standard materials based on chain growth polymerization. In the thiol‐ene reaction, a crosslinker with at least two thiol groups is necessary for network formation. Many currently used crosslinkers exhibit cytotoxic potential, are non‐biodegradable, or involve toxic chemicals and relatively complicated procedures in their synthesis, thus hindering their broader application. As an alternative, the use of a protein (fibrinogen gamma chain, FGG) recombinantly expressed in *Escherichia coli* was investigated. The FGG is part of the multimeric fibrinogen involved in hemostasis. This protein complex is stabilized by disulfide crosslinking. This presence of cysteines in the sequence makes FGG a promising candidate as a thiol donor in thiol‐ene reactions. It was shown for the first time, that a cysteine‐containing protein expressed in *E. coli* was capable of forming hydrogels with norbornene functionalized gelatin. An increase in FGG concentration led to higher gel stiffness and a decrease in the swelling ratio. Furthermore, the material exhibited cell adhesive properties and biocompatibility. Overall, a proof‐of‐principle could be achieved, opening up the use of recombinant proteins without further modifications as crosslinkers in thiol‐ene based hydrogels, providing a cost‐effective, safe, and scalable material source.

## Graphical abstract

Fibrinogen gamma chain (FGG) was recombinantly expressed in *E. coli* to generate a recombinant protein containing free thiol groups from cysteine. The expressed protein was used to crosslink norbornene functionalized gelatin providing a proof‐of‐principle of this novel crosslinker type and the formed hydrogels were characterized for their stiffness, mass swelling ratio and cellular adherence.

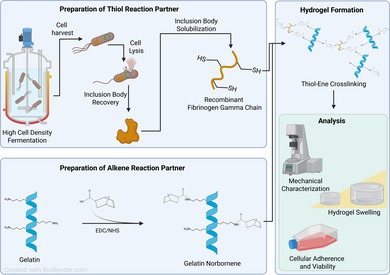

**Abbreviations**
DNB: defined non‐inducing broth
DO: dissolved oxygen
DTT: dithiothreitol
EDC: 1‐ethyl‐3‐(3‐dimethylaminopropyl)carbodiimide
EDTA: ethylenediaminetetraacetic acid
FGG: fibrinogen gamma chain
GelMA: gelatin methacrylate
GelNB: gelatin‐norbornene
hAD‐MSCs: human adipose‐derived mesenchymal stromal cells
LAP: lithium‐phenyl‐2,4,6‐trimethylbenzoylphosphinat
MES: 2‐(*N*‐morpholino)ethanesulfonic acid
MOPS: 3‐(*N*‐morpholino)propanesulfonic acid
NHS: *N*‐hydroxysuccinimide
OD_600_
: optical density at 600 nm
SDS‐PAGE: sodium dodecyl sulfate polyacrylamide gel electrophoresis
TNBS: 2,4,6‐trinitrobenzene sulfonic acid
TRIS: tris(hydroxymethyl)aminomethane

## 1 Introduction

Hydrogels are widely used in tissue engineering applications due to their tissue‐mimicking mechanical and bioactive properties. Many hydrogels are derived from natural materials; however, the formation of stable hydrogels at the conditions used in cell culture often necessitates covalent crosslinking. A widely applied strategy to form stable hydrogels is to modify the biological material with a reactive group that can be crosslinked in an easily controllable way. For this, the introduction of methacryloyl groups has been used for a variety of materials including collagen [1], gelatin [2, 3, 4], alginate [5, 6], hyaluronic acid [7, 8] and chitosan [9, 10]. Due to this broad application, methacrylation is currently regarded as a standard technique for the generation of stable, light‐crosslinkable hydrogels, with gelatin methacrylate (GelMA) being one of the most widely used materials.

However, alternative crosslinking chemistries have gained increased attention, as chain‐growth polymerization (such as methacrylate‐based crosslinking) has been shown to generate higher free radical concentrations, which can damage proteins and thereby compromise the viability of encapsulated cells [11]. Furthermore, methacrylate‐based chain growth polymerization is oxygen inhibited [12, 13]. This can cause longer processing times in additive manufacturing techniques during possible future applications in regenerative medicine, as the oxygen inhibition leads to longer exposure time for every layer, thereby increasing total fabrication time. In addition, prolonged incubation time in methacrylate‐based materials has been shown to cause adverse effects on cell viability [14], thus making an improved crosslinking mechanism desirable.

One promising alternative to methacrylate‐based hydrogels is the use of thiol‐ene chemistry for light‐induced crosslinking. Both crosslinking strategies can be applied using the same radical starters and machinery [14, 15], so a switch from methacrylate‐based crosslinking towards thiol‐ene based hydrogels does not require modifications in laboratory equipment. GelNB has been shown to exhibit faster polymerization reaction rates compared to GelMA [16]. Furthermore, by varying the thiol‐containing crosslinker, the properties of the resulting hydrogel can be adjusted by increasing crosslinking density [15, 17].

However, the requirement of multivalent thiols as a reaction partner also displays some downsides. A commonly used crosslinker, dithiothreitol (DTT), shows cytotoxic effects itself [14, 17, 18]. Other possible crosslinkers are thiolated macromolecules such as polyethyleneglycol (PEG) [15, 17, 19, 20], or proteins [14, 17, 21, 22]. These materials have no adverse effect on cell viability [14, 17]. However, the introduction of the thiol groups necessary for crosslinking requires additional chemical modification. This is usually done under inert atmosphere due to the reactive thiol group [15, 17, 21] complicating their synthesis and scale‐up. Other crosslinkers used in thiol‐ene based hydrogels are synthetic peptides carrying cysteine as a naturally occurring thiol group and often also providing other biological activities such as binding sites [23, 24, 25]. However, peptide synthesis involves several hazardous chemicals and is relatively time consuming with reaction times of up to 20 min per amino acid [26]. Our group recently reported the use of a laminin‐derived peptide containing two cysteines as a crosslinker of thiol‐ene hydrogels. While the peptide provided an environment suitable for neuronal stem cell proliferation, additional crosslinking with DTT was necessary to provide sufficient stability of the gels [27].

The choice of thiol‐providing reaction partner also influences the hydrogel properties. Higher functionality of the thiolated crosslinker leads to stiffer materials with reduced mass swelling ratios [15, 17]. Furthermore, the molecular weight and position of thiols within the molecule can influence material characteristics. Thiolated PEG of different molecular weights was shown to exhibit different stiffness and mass swelling ratios. Besides possible phase separation effects, this was partially attributed to the large distance between the thiol groups in the PEG with higher molecular weight, causing a lower stiffness and increased mass swelling ratio [17].

Other potential thiol‐crosslinkers are recombinant cysteine‐containing peptides or proteins. Disulfide bridge formation is mostly prevented in the cytoplasm of *Escherichia coli* [28, 29], possibly making it a suitable expression host for the production of cysteine‐containing proteins later used in thiol‐ene crosslinking. Furthermore, *E. coli* is a well‐studied expression host for recombinant proteins, as shown in numerous studies [30, 31].

Proteins containing cysteines can form disulfide bridges, which contribute to protein stabilization. One such example is fibrinogen, a blood plasma protein multimer composed of three pairs of individual polypeptide chains, whose structure is stabilized by multiple disulfide bridges [32]. This makes recombinant fibrinogen chains promising candidates as multivalent thiol‐containing molecules as a reaction partner in thiol‐ene hydrogels. Fibrinogen‐based materials are widely used in tissue engineering applications, as recently reviewed by Li et al. [33]. Here, fibrinogen provides valuable biological functions, including growth factor binding sites for fibroblast growth factor 2 [34], vascular endothelial growth factor [35] and interleukin 1β [36], as well as adhesion sites for different cell types including endothelial cells and platelets [37] immune cells [38] and fibroblasts [39]. Moreover, fibrinogen coatings demonstrate support of stem cell proliferation and differentiation [40]. Due to these properties and its extracellular matrix‐like structure, fibrinogen is a promising material for hydrogel formation. Thrombin‐mediated crosslinking produces fibrin hydrogels in a gentle crosslinking method that supports diverse tissue culture applications. Fibrin gels are capable of modulating immune response and accelerate skin wound healing by recruiting relevant cell populations [41], while fibrin gel coatings of ceramic matrices enhance osteogenic differentiation and bone formation [42], demonstrating wide‐range clinical potential for fibrin gels.

In this study, we adopted an alternative strategy for thiol‐ene hydrogel generation by expressing only the fibrinogen gamma chain (FGG) of the hexameric fibrinogen complex in *E. coli* . All three different fibrinogen chains (alpha, beta, and gamma) possess cysteines and participate in inter and intra‐chain disulfide bonds for final fibrinogen assembly [43, 44]. We selected the FGG chain for sole expression in *E. coli* , resulting in the production of a non‐glycosylated protein in inclusion bodies. FGG was chosen for its integrin binding sites, for its FGF‐2 binding site [45] and especially for its Factor XIII crosslinking site [46]. FGG introduces attractive biological activity into the thiol‐ene hydrogel while potentially enabling a subsequent method for stiffness control by additional enzymatic crosslinking with the transglutaminase factor XIII.

Given that the recombinantly expressed FGG contains 10 cysteine residues distributed along its sequence, we hypothesized that expressed FGG would present multiple accessible thiol groups that could be used to crosslink norbornene‐functionalized polymers by a thiol‐ene reaction. This would be the first description of recombinant proteins containing several cysteines as crosslinkers in thiol‐ene chemistry.

**FIGURE 1:**
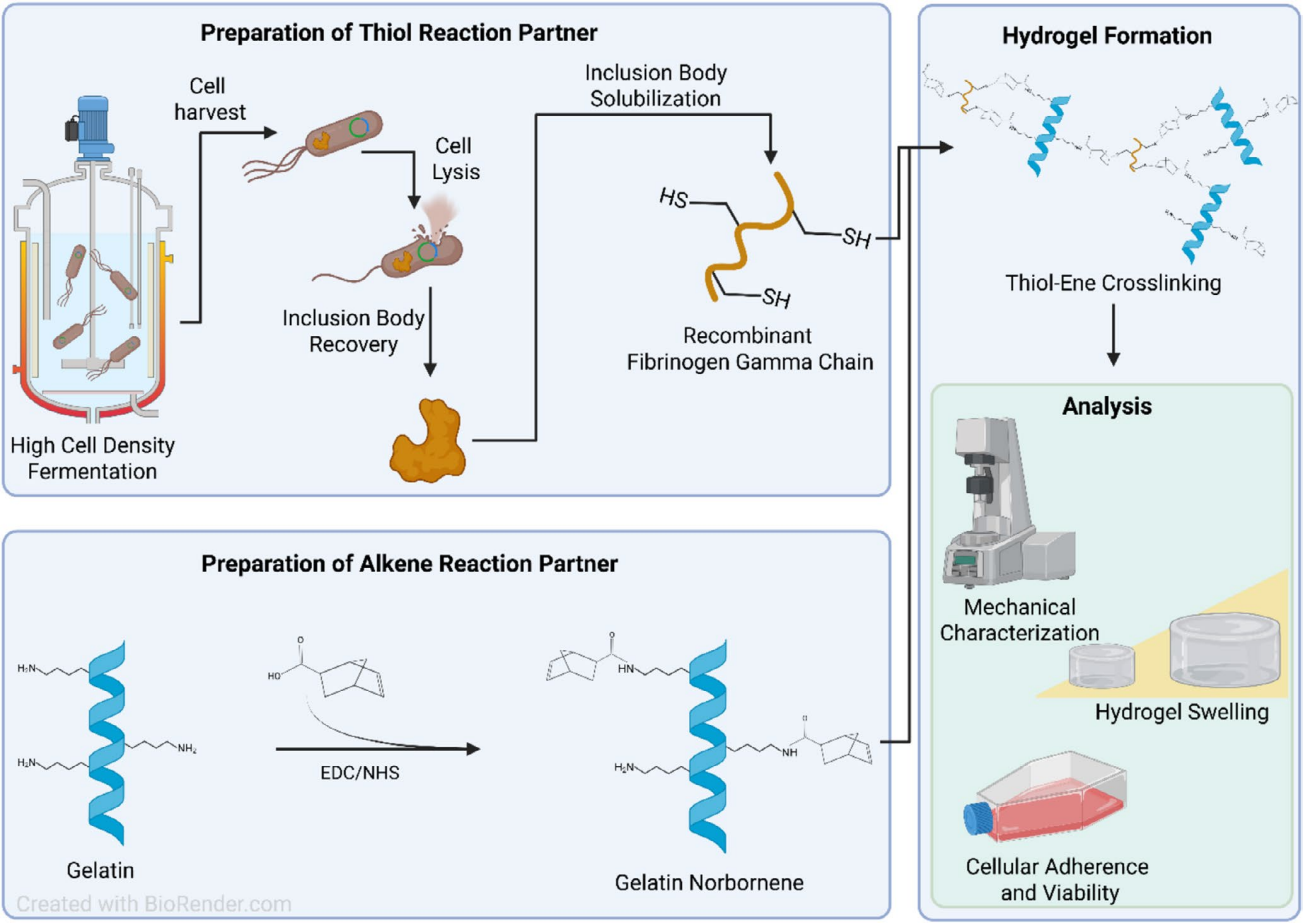
Graphical Abstract of the work conducted in this study. “Graphical abstract” created in BioRender. S, D. (https://BioRender.com/kvbu9i0) is licensed under CC BY 4.0 [47].

Based on this hypothesis, our specific objectives were: (1) to express and purify recombinant FGG and quantify its free thiol content, (2) to evaluate the ability of FGG to crosslink norbornene‐functionalized gelatin (GelNB) by rheological characterization, (3) to verify the involvement of FGG thiols in network formation by comparing free thiols before and after crosslinking and (4) to assess mass swelling and suitability of the resulting gels for cell culture as proof‐of‐principle for using recombinant cysteine‐rich proteins such as FGG to crosslink norbornene‐functionalized materials. The workflow is also depicted in Figure 1.

## 2 Material and Methods

All chemicals were purchased from Carl Roth, if not otherwise stated. Restriction enzymes were purchased from Thermo Fisher Scientific. All *E. coli* cultivations were performed with medium containing 100 μg mL^−1^ ampicillin. Carbic anhydride was purchased from BLD Pharmatec GmbH; 2,4,6‐trinitrobenzene sulfonic acid was purchased from Sigma‐Aldrich.

### 2.1 Cloning for Generation of the Empty Vector Control

The pET16b‐FGG vector was obtained from previous work [48]. The protein sequence of FGG is also given in the Supporting Information. For generation of a negative control strain, the pET16b vector backbone was amplified using the pET16b‐FGG vector as a template with the primers 5′‐GTTCTCGAGCGAAAGGAAGCTGAGTTGGC‐3′ and 5′‐CATCTCGAGCCGCTGCTGTGATGATGATG‐3′. The product was purified from the agarose gel with a Monarch gel extraction kit (New England Biolabs) following the manufacturers' instructions. The isolated DNA was cut using FastDigest XhoI, then purified using a QIAquick PCR purification kit (QIAGEN) and ligated using instant sticky end ligation mix (New England Biolabs) while transforming into Top10 *E. coli* (Invitrogen). Transformants were grown on Luria‐Bertani (LB) plates at 37°C for 16 h. Several colonies were picked and grown in 5 mL LB medium overnight at 37°C and 150 rpm. Plasmid DNA was isolated using a QIAprep spin miniprep kit (QIAGEN). The Isolated DNA sequence was verified by plasmid sequencing (Eurofins Genomics). A plasmid showing the correct DNA sequence was used for transformation of Tuner *E. coli* (Merck) for generation of the empty vector control strain.

### 2.2 Expression and Purification of Recombinant FGG From Inclusion Bodies

The expression of recombinant FGG was performed in a fed‐batch process as described earlier [49]. Defined non‐inducing broth (DNB) was prepared according to Li et al. [50]. Briefly, A 2 L Biostat A Plus was filled with 756 mL H_2_O and equipped with a dissolved oxygen (DO) and pH electrode as well as two Rushton impellers and subsequently autoclaved. Following the sterilization of the reactor, sterile stock solutions (20 mL MgSO_4_ [60 g L^−1^], 50 mL glucose·H_2_O [240 g L^−1^]), 200 μL Na_2_MoO_4_·2H_2_O [10.5 g L^−1^], 500 μL trace element solution (CoCl_2_·H_2_O [5 g L^−1^], MnCl_2_·4H_2_O [30 g L^−1^], CuCl_2_·2H_2_O [3 g L^−1^], H_3_BO_3_ [6 g L^−1^], Zn(CH_3_COO)_2_·2H_2_O [67.6 g L^−1^], Ethylenediaminetetraacetic acid (EDTA) [28.2 g L^−1^]), 20 mL (NH_4_)_2_HPO_4_ [200 g L^−1^], 100 mL KH_2_PO_4_ [133 g L^−1^], 20 mL citric acid [77.71 g L^−1^], 20 mL Fe(III)citrate [5.04 g L^−1^], 13.5 mL NaOH [200 g L^−1^] were added using a feed flask to fill the reactor with 1 L DNB. A pre‐culture was grown in 50 mL DNB medium overnight at 37°C and 150 rpm in a baffled Erlenmeyer flask. The bioreactor was inoculated to an optical density at 600 nm (OD_600_) of 0.1. The fermentation was performed at 37°C, and a DO was set to a minimum of 30%. DO was controlled by gassing with a constant flow rate of 1 L min^−1^ and increasing the stirring speed as well as supplementation of oxygen to the ingas stream. The pH was set to 7 and controlled using 3 M HCl and 25% Ammonium solution. Following the depletion of glucose in the batch phase observed by a sudden decrease in oxygen demand, a feed (129.89 g glucose·H_2_O, 100 μL trace element solution 40 μL Na_2_MoO_4_ solution, 1.181 mL Fe(III) citrate solution, 4 mL MgSO_4_ solution, 200 μL ampicillin solution filled to a total volume of 200 mL with H_2_O) was set up with a flow rate of 7.2 mL h^−1^. IPTG was added at 0.5 mM 7.5 h after initiation of the feed for FGG expression, and the protein was expressed for 4 h (Induction 6 h after feed start for the empty vector and expression for 5.5 h). The culture was harvested, and cells were obtained by centrifugation at 4200 g for 30 min. The pellet was dissolved in lysis buffer (3‐(*N*‐morpholino)propanesulfonic acid (MOPS) 50 mM, NaCl 150 mM, EDTA 1 mM) at 20 mL buffer per gram pellet and centrifuged for 30 min at 4200 g and 4°C. The supernatant was discarded, and the pellet was dissolved again in lysis buffer at 20 mL buffer per gram pellet. The cells were lysed using a high‐pressure homogenizer at 9000 psi in 8 repetitions. The lysed cells were centrifuged at 4200 g for 30 min. The pellet was resuspended in 4 mL g^−1^ pellet washing buffer (tris(hydroxymethyl)aminomethane (TRIS)‐HCl 50 mM, NaCl 100 mM, urea 1 M, EDTA 1 mM, Triton 1% (v/v), pH 8) using an ultra turrax and centrifuged as described above. The washing was repeated three times before the pellet was resuspended in 4 mL solubilization buffer (TRIS–HCl 50 mM, NaCl 100 mM, urea 5 M, pH 8) per gram of pellet. The solution was frozen overnight at −20°C and centrifuged again at 4200 g for 1 h.

### 2.3 Ammonium Sulfate Precipitation

For precipitation of the solubilized protein, 30% (w/v) ammonium sulfate was added to the protein solution and centrifuged at 4200 g for 15 min. The pellet was then resuspended in the same volume of solubilization buffer as before. The empty vector control was precipitated accordingly.

### 2.4 SDS‐PAGE

Samples taken during fermentation were all dissolved in lysis buffer to a target OD of 10 and cells were lysed by ultrasonication. For SDS‐PAGE 10 μL 4× Laemmli buffer was mixed with 30 μL sample and incubated at 95°C for 5 min. 5 μL samples were loaded per lane. The gel was run at 120 V for 10 min and subsequently at 180 V for 30 min. 1.5 μL pre‐stained protein standard (Thermo Fisher Scientific) was added to one lane for size comparison. The gels were stained with Coomassie overnight for visualization of the protein bands. For densitometric protein quantification, bovine serum albumin was added in different concentrations as a standard. Quantification was performed using the GelAnalyzer software (GelAnalyzer 23.1.1, www.gelanalyzer.com).

### 2.5 Functionalization of Gelatin

The synthesis of norbornene functionalized gelatin (GelNB) was performed as described by Göckler et al. [14]. Briefly, 368 mg 5‐Norbornene‐2‐carboxylic acid per gram of gelatin (2 equiv. compared to primary amine content in gelatin) was dissolved in 10 mL 2‐(N‐morpholino)ethanesulfonic acid (MES) buffer 0.5 M (pH 6), and 4 equiv. EDC (1.02 g) and 2 equiv. NHS (0.31 g) were added to activate the norbornene at 50°C for 15 min. 5 g gelatin (Type A, 300 bloom, Sigma‐Aldrich) were dissolved in 10 mL MES buffer per gram gelatin at 50°C. The dissolved gelatin was added to the activated norbornene, and the pH was adjusted to pH 7.5 with NaOH. The reaction was allowed to proceed overnight before the mixture was centrifuged and dialyzed for 4 days against H_2_O using dialysis tubes with a molecular cut‐off weight of 14 kDa (Spectra Por). After dialysis, the GelNB was lyophilized and stored at −20°C until use.

GelNB synthesized without the use of EDC and NHS was prepared based on the procedure described by Mũnoz et al. [15] with some modifications that were based on the optimized synthesis protocol for GelMA published by Shirahama and Lee [4]. The buffer system was changed from PBS to 0.25 M carbonate–bicarbonate (CB) buffer with a pH of 9. Furthermore, the amount of carbic anhydride was reduced from 20% (w/v) to match the same equivalents of 5‐Norbornene‐2‐carboxylic acid used in the EDC‐NHS based GelNB synthesis. In brief, gelatin was dissolved at 10% (w/v) in 0.25 M CB buffer at 50°C under stirring. The pH was adjusted to 9, and 0.13 g carbic anhydride per gram gelatin was added. After 30 min of reaction, the pH was adjusted to 9 with 1 M NaOH, and the reaction was performed for 6 h. The synthesis mixture was dialyzed, dried, and stored as described above. The reagent ratios and resulting DoF for both synthesis procedures are also given in the Supporting Information in Table S1.

### 2.6 2,4,6‐Trinitrobenzene Sulfonic Acid Assay

To determine the DoF of the GelNB synthesized, TNBS assay was performed. For this, Gelatin and GelNB were dissolved in 500 μL of 0.1 M CB buffer (pH 9) at 0.4 mg mL^−1^. A total of 500 μL glycine standards were prepared at 10, 5, 2.5 and 1.25 μg mL^−1^. A total of 500 μL 0.01% TNBS in 0.1 M CB buffer were added to each sample and incubated at 37°C for 2 h. The reaction was terminated by the addition of 500 μL 10% sodium dodecylsulfate and 250 μL of 1 M HCl. The absorbance was measured at 335 nm. Primary amine concentration was determined by comparison with a glycine standard and the DoF was calculated as the difference between the amine content in gelatin and GelNB divided by the amine content in gelatin.

### 2.7 Nuclear Magnetic Resonance Spectroscopy

Correct functionalization of the materials was verified by NMR spectroscopy. For this, 20 mg of unfunctionalized materials, as well as 20 mg of the material functionalized with norbornene groups by the different synthesis procedures were dissolved in 1 mL D_2_O. ^1^H‐NMR spectra were recorded on an Ascend 600 MHz NMR magnet with an Avance Neo console (Both Bruker). NMR spectra were processed using the MestreNova software (14.2.) (Mestrelab Research).

### 2.8 Quantification of Thiols With Ellman's Assay

Free thiol groups were detected by an Ellman's assay. A total of 50 μL of 10 mM Ellman's reagent solution were mixed with 2.5 mL reaction buffer (0.1 M sodium phosphate, 1 mM EDTA, pH 8) and 250 μL sample were added. The reaction was allowed for 15 min before 200 μL of the reaction were transferred to a 96‐well plate and the absorbance was measured at 412 nm. Thiol concentration was determined by comparison with a cysteine standard at 1.6 , 0.8, 0.4, 0.2, and 0.1 mM.

For quantification of free thiols in gels, FGG solution was diluted 4‐fold with solubilization buffer, and 250 μL were used for dissolving GelNB at 5% (w/v) with 0.1% Lithium‐Phenyl‐2,4,6‐trimethylbenzoylphosphinat (LAP), and the gels were cured in a 24 well plate in a UV chamber (Vilber Biolinker) with 1.2 J cm^−1^. The remaining assay was performed as described above inside the well with the crosslinked gel. The incubation time was extended to 6 h in the dark to allow diffusion of the reagents into the gel as described by Göckler et al. [14]. FGG solution with the same concentration also containing 0.1% LAP and the same UV dosage was used as a reference and set to 100%, while GelNB containing 0.1% LAP without FGG also exposed to the same UV dose was used as a negative control.

### 2.9 Rheology

Rheological measurements were performed on a MCR302 rheometer (Anton Paar). Storage and loss modulus were recorded at 0.1% deformation at 1 Hz. All measurements were performed at 37°C in the presence of 0.1% LAP. The samples were measured for 6 s, before the sample was crosslinked with UV light (365 nm) for 250 s followed by 40 s of measurement without UV. If not stated otherwise, all rheological measurements were performed using the above‐mentioned UV exposure.

#### 2.9.1 Comparison of FGG to Empty Vector Control Before Precipitation

For comparison with the empty vector control before additional purification by precipitation, FGG was diluted after being dissolved in solubilization buffer to a concentration of 2.5 and 5 mg mL^−1^. The empty vector control was diluted using the same ratios to achieve an equivalent concentration compared to the FGG samples that are indicated by the corresponding FGG concentration at this dilution. GelNB prepared by EDC/NHS coupling was dissolved at 5% (w/v) in these samples, as well as in H_2_O and solubilization buffer. GelNB prepared by reaction with carbic anhydride was dissolved in H_2_O and an empty vector control solution corresponding to the dilution for 5 mg mL^−1^ in FGG samples.

#### 2.9.2 Comparison of FGG to Empty Vector Control After Precipitation

After the additional precipitation step, GelNB was dissolved at 5% (w/v) in solubilization buffer containing 1.75 and 3.5 mg mL^−1^ FGG and at 10% (w/v) in solubilization buffer containing 1.75 mg mL^−1^ FGG. GelNB was also dissolved at 5% (w/v) in solubilization buffer with the empty vector control diluted in the same ratios to achieve equivalent samples for 1.75 and 3.5 mg mL^−1^ furthermore, 5% GelNB was dissolved in one undiluted sample of the empty vector control equivalent to 7 mg mL^−1^ of the FGG sample. 10% GelNB (w/v) was dissolved in the empty vector control at a concentration equivalent to 1.75 mg mL^−1^ FGG. Rheological crosslinking experiments were performed as stated above.

#### 2.9.3 Comparision of FGG to DTT With Matched Thiol‐Concentration

For comparison with DTT, GelNB was dissolved at 5% and 10% (w/v) with different concentrations of FGG. DTT was added to match the thiol concentration of the different FGG concentrations as determined by Ellman's assay. 5% GelNB (w/v) was dissolved in solubilization buffer containing either FGG or DTT with a thiol concentration of 0.4, 2, and 4 mM. 10% GelNB (w/v) was dissolved in solubilization buffer containing 0.4 and 2 mM thiols from FGG or DTT. Rheological measurements were performed as described above.

### 2.10 Hydrogel Washing and Urea Detection

A total of 200 μL of 5% GelNB with different FGG concentrations and 0.1% LAP were cured in a Vilber Biolinker (Vilber) with 1.2 J cm^−2^ in a 24‐well plate (Sarstedt). A metal mesh was placed on top of the hydrogel to prevent damaging the hydrogel while exchanging the water in the well. The water was exchanged every 10 min for the first hour; then, water exchange times were gradually increased, reaching water exchange twice a day. Urea was detected on a Chromomaster (Hitatchi) high‐performance liquid chromatography system equipped with a Synergi 4u Fusion‐RP 80A column (Phenomenex). H_2_O with 2% ethanol was used as a mobile phase with a flow rate of 0.8 mL min^−1^. A total of 10 μL of the sample was injected, and absorbance was measured at 202 nm.

### 2.11 Hydrogel Swelling

For swelling, 5% GelNB were dissolved with FGG at different concentrations, as well as 0.1% LAP. For comparison, samples with DTT matching the thiol concentrations of the FGG samples were prepared in parallel. A total of 200 μL were cured on a glass slide in a UV chamber (Vilber Biolinker) with 1.2 J cm^−1^ and swollen in 30 mL H_2_O for 24 h. Gels were weighed in their swollen state, lyophilized and subsequently their dry weight was determined. The swelling ratio was determined by the mass of the swollen gel minus the weight of the dried gel divided by the weight of the dried gel as given by the formula below.

$$\text{Swelling ratio}=\frac{\left(\text{weigh}t_{\text{swollen}}-\text{weigh}t_{\mathrm{dry}}\right)}{\text{weigh}t_{\mathrm{dry}}}$$

### 2.12 Cell Culture

FGG was thawed and homogenized by vigorous pipetting. Insoluble protein was removed by centrifugation at 13000 rpm for 5 min. GelNB was dissolved at 5% (w/v) in the FGG supernatant at 37°C and LAP was added to a final concentration of 0.1%. The solution was sterilized by filtration through a 0.2 μm filter. A total of 200 μL of the material were filled to a 24‐well plate and crosslinked in a UV chamber (Vilber Biolinker) with 1.2 J cm^−2^. The gels were transferred to a 6‐well plate and washed with H_2_O for 3 days with the water repeatedly exchanged to wash out urea from the solubilization buffer. The gel was cut in small pieces and the pieces were transferred to a 96‐well BIOFLOAT plate (Sarstedt). Primary human adipose derived mesenchymal stromal cells (hAD‐MSCs) were obtained from the works of Schmitz et al. [51]. There, the cells were isolated from adipose tissue received from abdominoplasty. The tissue was minced using sterile scissors and digested with collagenase solution at 4°C over‐night. After washing with Hank's balanced salt solution, the cells were recovered by centrifugation and subsequent seeding and adherence to cell culture plates. All patients gave their informed consent as approved by the Hannover Medical School's Institutional Review Bord under the reference number 3475‐2017. A total of 5000 hAD‐MSCs were seeded per each well in 150 μL alpha MEM (Gibco, Thermo Fisher Scientific) containing 10% human serum (cc.Pro, Germany) and 0.05 mg mL^−1^ gentamycin (Merck KGaA) and incubated at 37°C and 5% CO_2_ in the IncuCyte S3 imaging system (Sartorius). Time‐laps images were taken at 20 min intervals. After 2 days of culture, live/dead staining was performed using 7 μM Calcei‐AM and 1.5 μg mL^−1^ propidium iodide. Fluorescence images were taken using the IncuCyte S3 imaging system.

### 2.13 Statistical Analysis

The influence of crosslinker concentration and GelNB concentration on material stiffness was analyzed by two‐way ANOVA using the statsmodels library [52]. Comparison between different crosslinkers was performed by unpaired *t*‐test using the scipy library [53]. Plots were generated using the matplotlib library [54], box plots were generated using the seaborn library [55]. In statistical analysis, a *p*‐value < 0.05 was considered significant. All data were measured in triplicates if not stated otherwise and originate from a single batch of FGG.

## 3 Results and Discussion

### 3.1 Expression of FGG and Empty Vector Control

**FIGURE 2:**
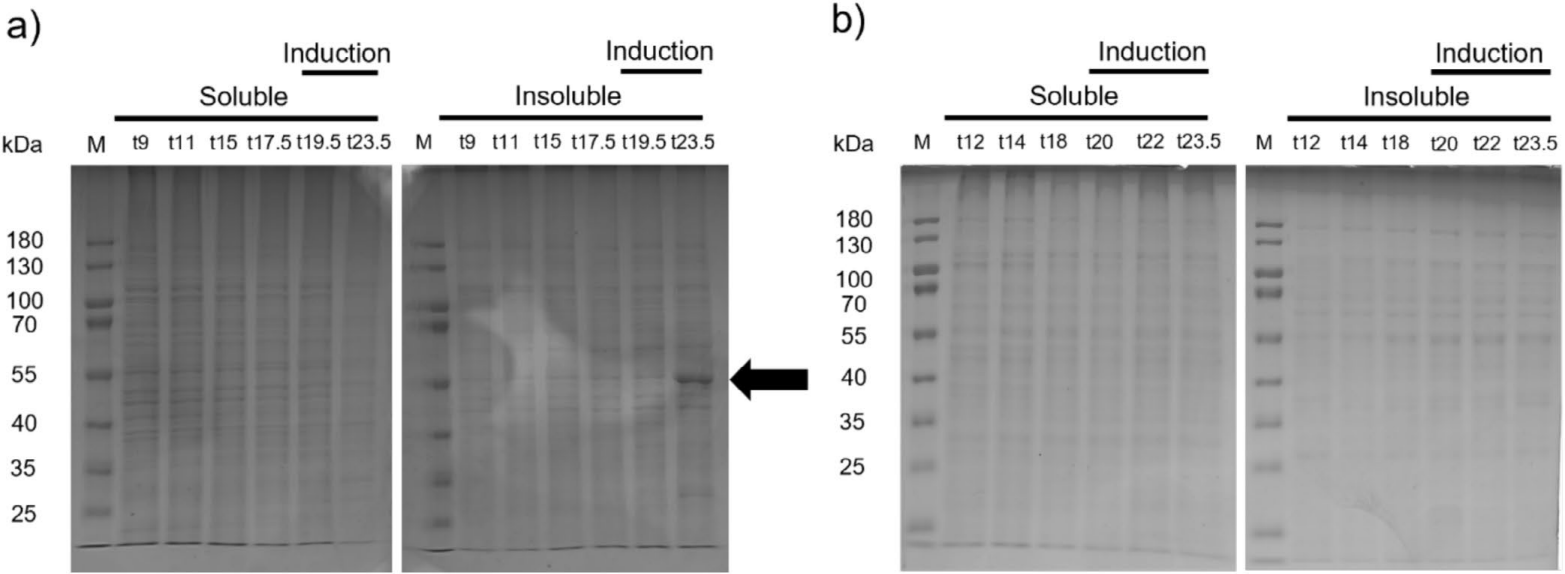
(a) SDS‐PAGE of soluble and insoluble protein fraction from lysed cells taken during the FGG expression. (b) SDS‐PAGE of the soluble and insoluble protein fraction of lysed cells taken during the cultivation of the empty vector.

Initially, FGG was expressed in *E. coli* as a possible reaction partner in thiol‐ene hydrogels. Protein expression was controlled by the IPTG inducible T7 promoter. The course of the fermentation is shown in the Supporting Information in Figure S1. The cells were grown in a batch phase until an oxygen spike was detected marking the end of the batch phase. Then, a feed was started to further increase the biomass. Protein expression was induced 4 h after the feed was started. A cultivation of the empty vector control was performed in an analogous manner, but in this case the culture was harvested earlier compared to the FGG expression due to an observed reduction in cell density. As seen in Figure 2a, FGG was expressed in the insoluble fraction at an apparent molecular weight of around 55 kDa. No comparable protein band was detected in the empty vector control (see Figure 2b). The band at 55 kDa was identified as FGG by Western blot in a previous work [48].

### 3.2 Crosslinking of GelNB With FGG and Empty Vector Control Before Precipitation

To investigate the ability of FGG to participate in light induced thiol‐ene reactions, FGG and an empty vector negative control were used for crosslinking reactions with GelNB. FGG was recombinantly expressed in the insoluble protein fraction of *E. coli* , while the empty vector control was obtained from the insoluble fraction of *E. coli* lysate without the FGG expression cassette. GelNB was synthesized as an alkene bearing material for thiol‐ene crosslinking using EDC‐NHS coupling of a norbornene group to gelatin. ^1^H‐NMR spectra recorded for GelNB are shown in Figure S2.

In a first trial, FGG and the empty vector control were directly used in crosslinking experiments following their solubilization in the presence of high urea concentrations. FGG had a concentration of 10 mg mL^−1^ determined by SDS‐PAGE densitometry, while almost no protein was detectable for the empty vector control. Ellman's assay showed a concentration of 3.39 mM thiol groups for FGG, while thiol concentration was below 0.1 mM for the empty vector control. To examine the effect of solubilization buffer alone during crosslinking, GelNB was also crosslinked when dissolved in H_2_O and solubilization buffer, without an additional reaction partner.

**FIGURE 3:**
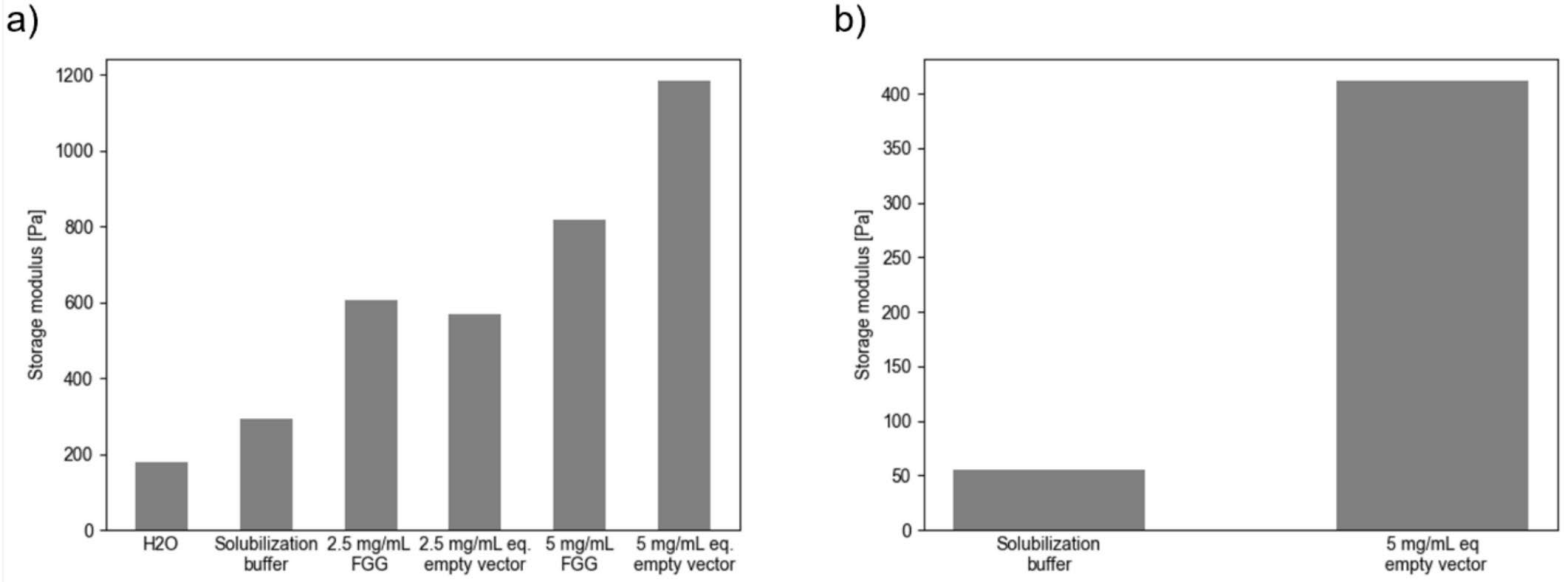
(a) Storage moduli measured for GelNB prepared by EDC‐NHS based synthesis for 5% GelNB (w/v) without additional crosslinkers, as well as with FGG and empty vector control at different concentrations. (eq. in empty vector control means equivalent dilutions compared to FGG, as equal protein concentration nor thiol concentration could be achieved); (b) Storage moduli for gels with 5% (w/v) of GelNB prepared by anhydride‐based synthesis as well as for gels composed of 5% GelNB with empty vector control. Samples with FGG and GelNB from anhydride synthesis *n* = 3, average values plotted, remaining samples *n* = 1.

As seen Figure 3a, GelNB polymerizes in the absence of a thiol‐bearing reaction partner in H_2_O as well as in solubilization buffer. GelNB alone reached a storage modulus of around 200 Pa in H_2_O and around 300 Pa in solubilization buffer. For FGG and the empty vector controls, a dose‐dependent increase in storage modulus was observed. While at 2.5 mg mL^−1^ FGG and the equivalent empty vector control both reached a stiffness of around 600 Pa, at 5 mg mL^−1^ FGG had a higher stiffness of 800 Pa, which is lower than the empty vector at that concentration reaching above 1100 Pa. As those values are higher than the ones observed in the negative control of GelNB without a reaction partner, it could be concluded that crosslinking is in fact happening between GelNB and the *E. coli* lysate. As the detected thiol concentration for the empty vector control was lower compared to FGG while the empty vector control reached comparable to higher stiffness, it was assumed that crosslinking is dominated by a side reaction and does not solely originate from a thiol‐ene reaction. Norbornene groups have been shown to undergo homopolymerization by reaction of alkenes in the absence of thiols before [56]. In our study, the self‐crosslinking of GelNB in the absence of a thiol seemed to be dependent on norbornene groups, as GelNB underwent gel formation during UV polymerization with a photoinitiator, without the presence of a thiol crosslinker (See Figure S4), while unmodified gelatin did not (See Figure S3c). As a result, norbornene groups might also react with different functional groups in cell lysates.

Another possibility considered was that residuals from EDC‐NHS coupling remaining in the GelNB by EDC‐NHS activation of 5‐Norbornene‐2‐carboxylic acid could react with the cell lysate, leading to unintended crosslinking. To investigate this possibility, GelNB was prepared by reacting gelatin with carbic anhydride completely avoiding EDC and NHS in the reaction (For ^1^H‐NMR spectrum see Figure S1c). Nevertheless, as seen in Figure 3b, GelNB synthesized by reaction with anhydride also showed higher stiffness in the presence of the empty vector control compared to the GelNB prepared by anhydride synthesis crosslinked in the absence of any additional reaction partner. As almost no thiols were detectable for the empty vector control and no EDC‐NHS was present in this GelNB batch, crosslinking must arise from the reaction of norbornene with alternative groups in the lysate. Indeed, even though the reaction mechanism of GelNB with *E. coli* lysate (shown to not contain significant thiols) would be interesting for future studies, it was not further investigated in this work. This was mainly due to the focus of FGG dependent crosslinking and due to the scarcity of material produced in small scale fermentations. It has to be noted that GelNB prepared by reaction with EDC and NHS as well as GelNB prepared by anhydride‐based synthesis showed slightly varying DoF. Therefore, the difference in stiffness of GelNB prepared by EDC/NHS coupling and by reaction with anhydride that have been crosslinked in solubilization buffer in the absence of any additional crosslinker as well as containing 5 mg mL^−1^ equivalent empty vector are not directly comparable regarding their stiffness. Since DoF has been shown to influence hydrogel stiffness [14], this might cause some variation in stiffness that could not be interpreted as different crosslinking efficiencies between the different tested materials. Furthermore, the EDC‐NHS coupling itself can cause interchain protein crosslinks in gelatin [57], effectively increasing the crosslinking density. The partial crosslinking of gelatin by EDC‐NHS was further supported by gel formation observed when the solubilized gelatin was added to the norbornene‐EDC‐NHS mixture during GelNB synthesis.

### 3.3 Crosslinking of GelNB With FGG and Empty Vector Control After Precipitation

After it became apparent that *E. coli* lysate influences GelNB crosslinking, additional protein purification was considered crucial to further investigate the suitability of FGG to serve as a thiol‐donor on thiol‐ene crosslinking. As the solubilized FGG had a protein concentration of 10 mg mL^−1^ determined by gel densitometry, while the empty vector contained almost no protein and the reaction did not seem to arise from thio‐ene reaction, it was assumed that mainly non‐protein contaminants caused the crosslinking in the empty vector control. Therefore, precipitation with ammonium sulfate before crosslinking experiments was performed in an attempt to remove non‐protein contaminants in the next experiments. Ellman's assay for FGG revealed a thiol concentration of 8.4 mM following precipitation, while gel densitometry gave a FGG concentration of 7 mg mL^−1^. Interestingly, the thiol concentration after purification of FGG was higher compared to the sample measured before precipitation (3.39 mM). As ammonium sulfate is not known to reduce disulfide bonds and the material showed visible signs of aggregation after precipitation, this increase is most likely caused by light scattering effects of aggregates in the sample. While the influence of aggregates from FGG in the measurement was minimized by sedimentation and use of the supernatant for thiol quantification, their influence might not be completely excluded. Therefore, Ellman's assay might overestimate the thiol concentration of FGG. The Ellman's assay was not repeated for the empty vector control as the thiol concentration was below the detection limit for the non‐treated sample before precipitation.

**FIGURE 4:**
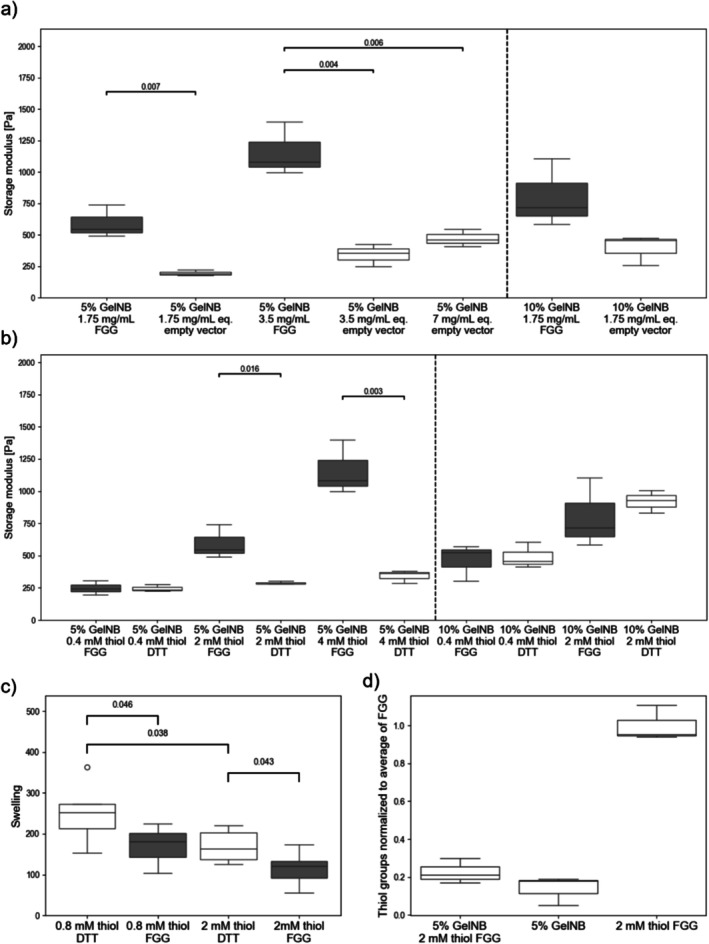
(a) Storage modulus recorded after UV curing of GelNB at 5% and 10% (both (w/v)) with different concentrations of FGG and matching with equivalent (eq.) concentrations of the empty vector control after precipitation. (b) Storage modulus recorded after UV curing of different FGG concentrations with 5% and 10% GelNB (both (w/v)) compared to DTT with matched thiol concentration. (c) Mass swelling ratio of hydrogels prepared from 5% GelNB (w/v) with FGG and DTT with matched thiol concentrations. (d) Thiol content of 5% GelNB crosslinked with FGG at 2 mM thiol concentration, as well as 5% GelNB (both with 0.1% LAP) normalized to FGG with 2 mM thiol concentration. (a), (b) and (d) *n* = 3; (c) *n* = 6.

Stiffness of GelNB at 5% and 10% (both (w/v)) crosslinked with different empty vector control and FGG concentrations are shown in Figure 4a. Two‐way ANOVA revealed that empty vector concentration and GelNB concentration both had a significant effect on hydrogel stiffness. This might indicate that precipitation did not remove all cell residuals involved in crosslinking. Also, two‐way ANOVA showed that increased FGG concentrations significantly increased hydrogel stiffness. An increase in GelNB concentration did not increase the hydrogel stiffness for FGG. However, FGG solubility was problematic, especially at 10% GelNB concentrations, which might reduce the hydrogel stiffness as a result of protein precipitating from the solution and thereby sequestering reactive groups together with their polymeric chains from the crosslinking reaction. In GelNB crosslinked with thiolated PEG of high molecular weight, phase separation has been shown to reduce hydrogel stiffness by impairing network formation [17]. However, phase separation has also been deliberately used in hydrogel formation as a structuring technique. Porous gelatin structures with defined pore sizes were formed by phase separation during freezing of gelatin gels prior to freeze drying. Control over the pore size was achieved by tuning of the temperature and time for freezing [58]. The preparation of microporous scaffolds was further achieved by liquid–liquid phase separation during crosslinking of thiol‐ene based systems, and the resulting difference in pore size affected cellular response in the generated material [59]. The creation of porous materials by phase separation was also combined with different additive manufacturing techniques to integrate macroscopical and microstructuring effects [60]. In our study, phase separation caused by protein precipitation reduced hydrogel stiffness, but this effect might also be employed for micropatterning just as has been described by others above.

When comparing FGG and the empty vector control at 5% GelNB concentration, 1.75 and 3.5 mg mL^−1^ FGG were more effective at hydrogel crosslinking than the equivalent empty vector control concentration, seen by significantly stiffer hydrogels obtained from FGG crosslinked samples. 5% GelNB crosslinked with 3.5 mg mL^−1^ also resulted in significantly stiffer hydrogels when compared with the empty vector control, corresponding to 7 mg mL^−1^. This indicates that FGG is involved in crosslinking of thiol‐ene gels following ammonium sulfate precipitation and is more effective in crosslinking than the empty vector control alone. Empty vector control and FGG alone did not form hydrogels during rheological experiments (See Figure S3a,b). Furthermore, FGG was able to crosslink GelNB at 5% (w/v), but not unmodified gelatin at the same concentration (Figure S3c,d).

### 3.4 Comparison of FGG to DTT as Thiol Donor in Thiol‐Ene Crosslinking

After showing a more effective crosslinking of GelNB by FGG compared to the empty vector control, the crosslinking of GelNB by FGG was compared to DTT, a common bifunctional crosslinker for thiol‐ene chemistry. The comparison of the hydrogel stiffnesses is shown in Figure 4b. The thiol concentrations in the FGG gels were matched with DTT concentrations, so the samples contained the same concentration of thiol groups. Increasing the FGG concentration strongly increased hydrogel stiffness in samples with 5% GelNB. In contrast, an increase in DTT concentration did only minorly increase stiffness in the samples with 5% GelNB. Crosslinkers with a higher number of thiol groups have been shown to produce stiffer gels before as they react with several GelNB chains [15, 17] which could explain the higher stiffness of FGG crosslinked gels compared to DTT due to higher functionality of the crosslinker. Despite this difference, an increase in thiol concentration relative to alkene groups led to stiffer gels. Increasing thiol concentration relative to alkenes at first increases crosslinking and gel stiffness before a reduction in stiffness is observed at even higher thiol concentrations [15, 16, 61]. As 4 mM thiol concentration used in this study is low compared to concentrations of 15 mM thiol from DTT used in other studies [15], it is most probable that crosslinking is limited by the low thiol concentration in the results presented here. Therefore, crosslinking could be further improved by an increase in thiol concentration. This could be either achieved by an increase in recombinant protein concentration or by the use of recombinant proteins with a higher number of cysteines. At 10% GelNB, increasing DTT concentration showed a stronger increase of the hydrogel stiffness, and the gels had a higher stiffness compared to gels of the same DTT concentration with 5% GelNB. This increase might arise from the higher protein content, resulting in more self‐crosslinking of the GelNB resulting from homopolymerization of the GelNB without involvement of additional reaction partners (for the homopolymerization of GelNB in rheological experiments see Figure S4). This, in combination with higher DTT concentration, can lead to more densely crosslinked networks between more gelatin strands. In contrast, at 10% GelNB concentration, gels with FGG showed a less pronounced increase in stiffness when the FGG concentration was increased. This might be a result of lower protein solubility in solutions containing 10% GelNB and also high FGG concentrations. It was not possible to dissolve 10% GelNB in a FGG solution containing 4 mM thiol in this study, so no values for 10% GelNB and FGG at this concentration are shown. This might indicate that protein solubility was already saturated in these samples. This incomplete dissolving of GelNB in FGG solutions might be already present in samples with lower FGG concentrations when GelNB concentration is increased to 10% but not macroscopically visible. This would result in lower effective GelNB concentration. A lower GelNB concentration would then cause reduced hydrogel stiffness [15, 27].

After comparing the stiffness of GelNB crosslinked either by FGG or DTT, the mass swelling ratios of hydrogels prepared using these two crosslinkers were investigated as a second important hydrogel property. As seen in Figure 4c, an increase in FGG concentration resulted in a decrease in mass swelling ratio. The same behavior was observed in GelNB crosslinked with matching concentrations of DTT. A significant decrease was found in gels crosslinked with 0.8 mM thiol from FGG and the same concentration of thiols from DTT, as well as between 2 mM thiol from FGG and the corresponding DTT concentration. Furthermore, increasing the DTT concentration significantly reduced the swelling. For FGG, the decrease in swelling between 0.8 mM thiol and 2 mM thiol could not be considered statistically significant (*p*‐value 0.0501) due to higher variation in the data. The decrease in swelling with increasing thiol concentration is explainable by higher crosslinking of the GelNB hydrogels. Dithiols result in higher swelling ratios compared to crosslinkers of higher functionality in some studies [15], but swelling seems to be highly dependent on the type and molecular weight of crosslinker and not only the amount of provided thiol groups [17]. As most studies compare DTT with thiolated polyethyleneglycol with large distances between the thiol groups, a direct comparison to these studies might be misleading, as in FGG, the thiols are spread throughout the protein chain. In fact, thiolated gelatin bearing multiple thiols spread throughout the molecule, which most closely resembles the overall structure of the FGG used in this study, resulted in a decreased water uptake capacity compared to crosslinkers such as DTT and thiolated PEG as a result of the higher thiol content per molecule [17]. Furthermore, when FGG is used instead of DTT as a reaction partner, more additional protein is added to the gel per thiol, which could additionally reduce the swelling ratio.

To verify that crosslinking of GelNB and FGG is based on a thiol‐ene reaction, free thiols in hydrogels after crosslinking were measured (See Figure 4d). It has been previously shown that an excess of thiol groups could be detected by performing the Ellman's assay with increased incubation time on gels [14]. Gels with 2 mM thiol from FGG showed a decrease in thiols of 80% compared to FGG of the same concentration after UV exposure in absence of GelNB, indicating that crosslinking is associated with the reaction of thiol groups.

While this reduction in thiol concentration indicated that thiols are involved in the reaction between FGG and GelNB as a proof‐of‐principle, possible impurities from the cell lysate still present in the FGG following precipitation could not be excluded to also react with the GelNB and thereby influence the hydrogel properties at this point. As the purity of FGG as well as the type of residues in the cell lysate causing the reaction with FGG could not be determined in this study, their possible influence remains unclear at this point and needs to be addressed in further studies. While we clearly showed that cysteines in FGG could be used in thiol‐ene reactions, the tendency to aggregate even in the presence of high urea concentrations hindered the use of FGG in crosslinking experiments, especially at thiol concentrations above 4 mM and also complicated FGG purification. For higher thiol concentrations, alternative proteins containing cysteines with higher solubility may be advantageous.

As proteins are not always stable in aqueous solutions, the FGG protein stability at 4°C was additionally analyzed. SDS‐PAGE of FGG samples stored for up to 13 days in solubilization buffer showed only a slight decrease in intensity of the protein band at 55 kDa (See Figure S5a). Besides protein stability, the concentration of free thiol groups necessary for thiol‐ene reaction was analyzed over a total time course of 4 days. Thiols from cysteine usually react by oxidation when stored for longer times in aqueous solutions [62]. As seen in Figure S5b in the Supporting Information, the thiol concentration normalized to the concentration at day 0 decreased linearly over the investigated timeframe and only 60% of the initial thiols were present after 4 days. This observation indicates instability of the free thiols in the recombinant protein over several days of storage. For future applications in hydrogels, it is therefore desirable to find storage conditions stabilizing the free thiols in the expressed proteins.

### 3.5 Removal of Urea From Hydrogels

**FIGURE 5:**
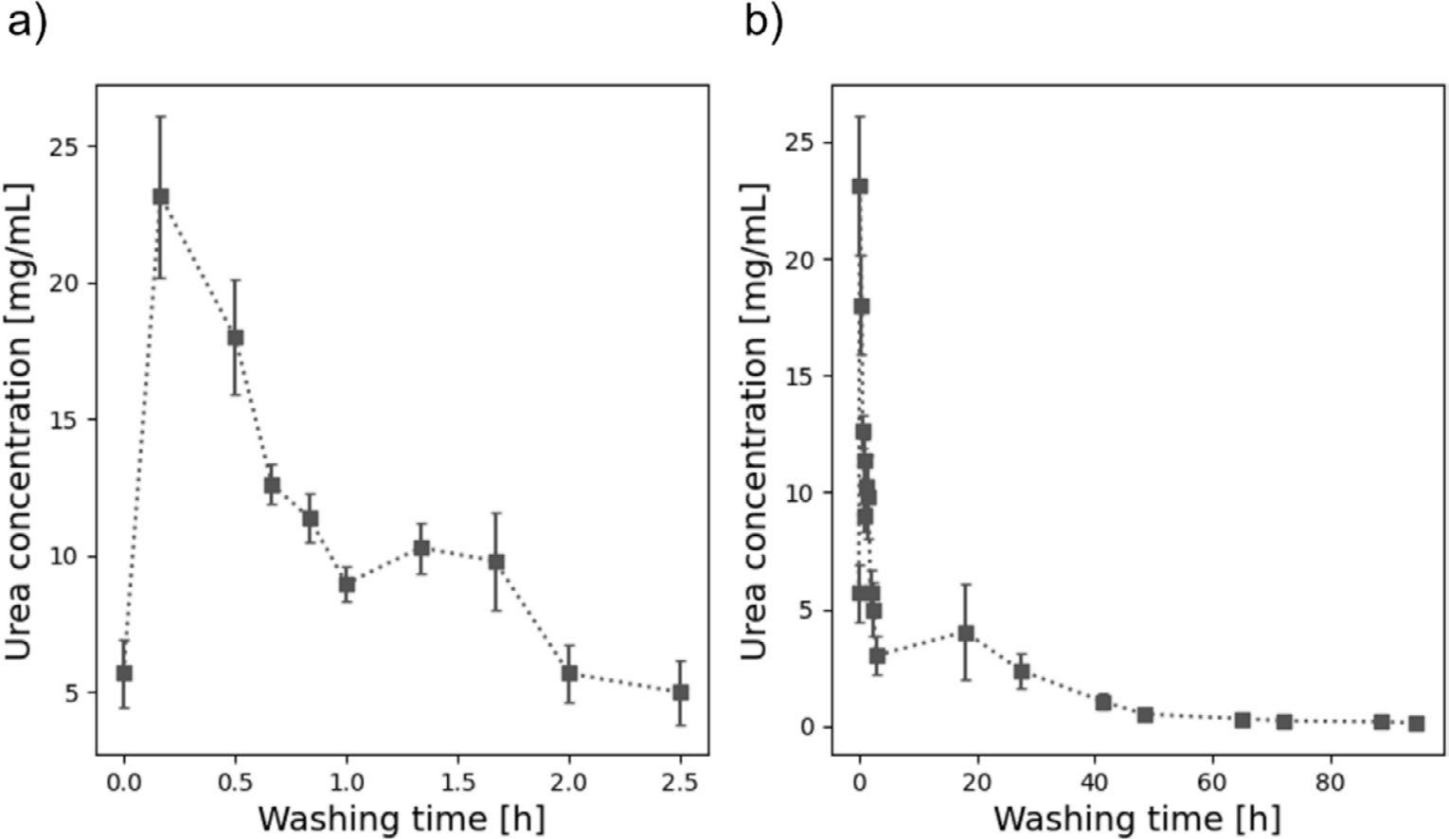
Urea concentration in the water surrounding the hydrogel over time. (a) Concentrations of the first 2.5 h, (b) concentration over 95 h. (The boxes display averages of each measurement; the line does not display measurements but rather gives guidance of the general trend) *n* = 5.

As the high urea concentration used for FGG solubilization might negatively influence the further application of the gels for example, during in vitro cultivation of cells on the material, removal of urea from the crosslinked gels was analyzed in a next step. As seen in Figure 5, urea could be detected in the surrounding liquid when the gels were incubated in H_2_O. The concentration of urea diffusing from the gel in the washing solution decreased sharply for the first few hours (see Figure 5a) and tended towards the detection limit after 48 h of incubation. Thus, it could be shown that urea can effectively be removed from GelNB‐FGG gels.

### 3.6 Cell Culture on GelNB‐FGG Hydrogels

**FIGURE 6:**
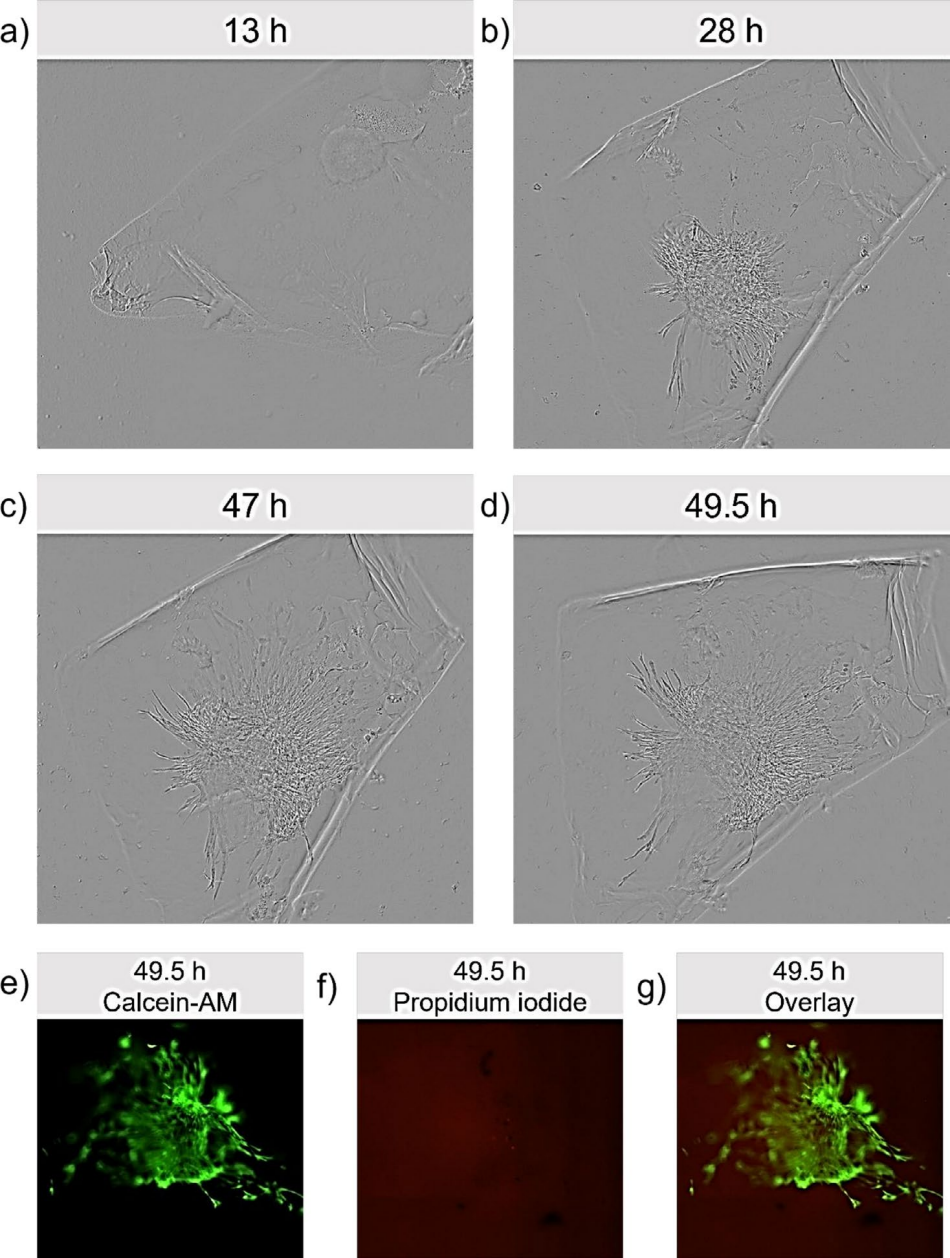
hAD‐MSC adhesion and viability on GelNB‐FGG hydrogels. (a–d) Phase contrast images of hAD‐MSCs adhering to GelNB‐FGG hydrogels between 13 and 49.5 h of culture; (e) live cells stained with Calcein‐AM (green channel); (f) dead cells stained with propidium iodide (red channel) and (g) overlay of Calcein‐AM and propidium iodide channels after 49.5 h.

In a last step, the general applicability of the GelNB‐FGG gels for cell culture applications was investigated. To evaluate the stability, biocompatibility, and cell‐adhesive properties of GelNB‐FGG hydrogels, primary hAD‐MSCs were seeded onto the crosslinked material. As shown in Figure 6a, cells initially formed a spheroid when not in direct contact with the hydrogel. Upon contact with GelNB‐FGG gel, the cells adhered and spread over the material, with this transition occurring between 13 and 49.5 h of culture (See Figure 6b–d). While this behavior clearly shows that crosslinking with recombinant FGG allows for cellular adhesion to the gels, it is not clear if the adhesion is also supported by the FGG itself or stems from the GelNB backbone. Cellular adhesion to GelNB crosslinked with multivalent thiols not providing cellular adhesion sites has been shown before, where GelNB crosslinked with DTT allowed for cell attachment visible by spreading of encapsulated cells over 13 days of culture [15]. Therefore, to investigate if FGG supports cell adhesion, FGG could be used to crosslink materials without cell binding sites as polyethylene glycol functionalized with norbornene groups in future experiments. In addition to observed cell spreading on the material, live/dead staining was performed after 49.5 h of culture to assess cell viability (Figure 6e–g). Calcein‐AM (green) labeled viable cells while propidium iodide (red) indicated dead cells. The results showed that the majority of cells remained viable at 49.5 h of culture, with only a few dead cells detected. By this, the general biocompatibility of the material could be shown, and at least after 3 days of washing, the high urea concentrations in the solubilization buffer did not lead to low cell viability. These short‐term experiments (49.5 h) confirm initial biocompatibility, cell adhesion, and material stability, ruling out cytotoxicity from *E. coli* residuals or urea buffer. Long‐term studies (7–30 days—standard for hydrogel biocompatibility ISO 10993‐5) remain essential to evaluate enzymatic degradation, matrix remodeling, and FGG‐specific bioactivity versus conventional crosslinkers.

Hydrogels prepared from gelatin by thiol‐ene crosslinking using different crosslinkers have shown high cell viability of encapsulated cells [14, 15, 27]. Furthermore, degradation products of GelNB hydrogels crosslinked with thiolated gelatin showed superior cell viabilities compared to DTT crosslinked gels [14]. This might indicate that protein based crosslinkers outperform other crosslinkers in regards to cell viability. However, as stated there, these differences could also be attributed to differences in gel compositions, especially to different photoinitiator concentrations used in that study. Besides cellular adhesion, the gel was shown to be stable for several days in solution during washing and cell culture, as well as for at least 2 days with cells seeded onto the surface, demonstrating the applicability of gels produced from norbornene functionalized materials crosslinked with recombinant cysteine containing proteins as FGG for 3D cell culture. However, the stability of the gels for prolonged time and their resistance to enzymatic degradation still needs to be evaluated. While the results presented here provide an initial proof‐of‐principle for the application of hydrogels composed of GelNB crosslinked with recombinant FGG in 3D cell culture, more detailed biological characterization including longer cultivation times of the material is necessary in the future. Future experiments must also compare FGG versus standard DTT/PEG crosslinkers over 7–30 days cultures to quantify degradation resistance, matrix remodeling, and possible FGG‐specific cell responses.

## 4 Conclusion

This study demonstrated that FGG could be recombinantly expressed in *E. coli* , presenting free thiol groups available for thiol‐ene based crosslinking. Recombinant FGG was thereby capable of crosslinking GelNB, with thiol groups in FGG being consumed during the reaction. The stiffness of GelNB hydrogels increased with increasing FGG concentration used for crosslinking, while the mass swelling ratio decreased. In comparison to DTT, FGG crosslinked gels exhibited higher stiffness and lower mass swelling ratio. The urea necessary for FGG solubilization could effectively be removed by hydrogel washing, and the washed gels were capable of supporting cell adhesion and migration. While providing an initial proof‐of‐principle for the applicability of recombinant proteins containing multiple cysteines as crosslinkers for light induced thiol‐ene crosslinking, the low solubility of FGG and therefore low thiol concentrations are limiting factors at this point.

## Funding

Funded by zukunft.niedersachsen, a funding programme of the Lower Saxony Ministry of Science and Culture and the Volkswagen Foundation as part of the “Matrix Evolution ‐ Hierarchisch strukturierte, bioinspirierte Matrizes” project.

## Conflicts of Interest

The authors declare no conflicts of interest.

## Supporting information


**Data S1:** Supporting Information.

## Data Availability

The data that support the findings of this study are available from the corresponding author upon reasonable request.
