# Supplementary material for: Recombinant Fibrinogen‐Gamma‐Chain as a Crosslinker of Thiol‐ene Hydrogels
Source: Biopolymers. 2026 Apr 3;117(3):e70097. doi: 10.1002/bip.70097 (PMC13049249; doi:10.1002/bip.70097)
Supplement: Supplementary file 1 — Data S1: Supporting Information. [file BIP-117-e70097-s001.docx]

Supporting Information (SI)

Recombinant fibrinogen-gamma-chain as a crosslinker of thiol-ene hydrogels

Domenic Schlauch^1^, Charlotte Selin Güler^1^, Marina Harzi^1^, Selin Kara^1^, Antonina Lavrentieva^1*^, Iliyana Pepelanova^1^

^1^ Institute of Technical Chemistry, Leibniz University Hannover, Callinstraße 5, 30167 Hannover, Germany.

^*^ Corresponding author

**E-mail:** lavrentieva@iftc.uni-hannover.de

## Protein sequence of recombinant Fibrinogen gamma chain

MGHHHHHHHHHHSSGHIEGRHMLEDIMYVATRDNCCILDERFGSYCPTTCGIADFLSTYQ

TKVDKDLQSLEDILHQVENKTSEVKQLIKAIQLTYNPDESSKPNMIDAATLKSRKMLEEIMKYEASILTHDSSIRYLQEIYNSNNQKIVNLKEKVAQLEAQCQEPCKDTVQIHDITGKDCQDIANKGAKQSGLYFIKPLKANQQFLVYCEIDGSGNGWTVFQKRLDGSVDFKKNWIQYKEGFGHLSPTGTTEFWLGNEKIHLISTQSAIPYALRVELEDWNGRTSTADYAMFKVGPEADKYRLTYAYFAGGDAGDAFDGFDFGDDPSDKFFTSHNGMQFSTWDNDNDKFEGNCAEQDGSGWWMNKCHAGHLNGVYYQGGTYSKASTPNGYDNGIIWATWKTRWYSMKKTTMKIIPFNRLTIGEGQQHHLGGAKQAGDV


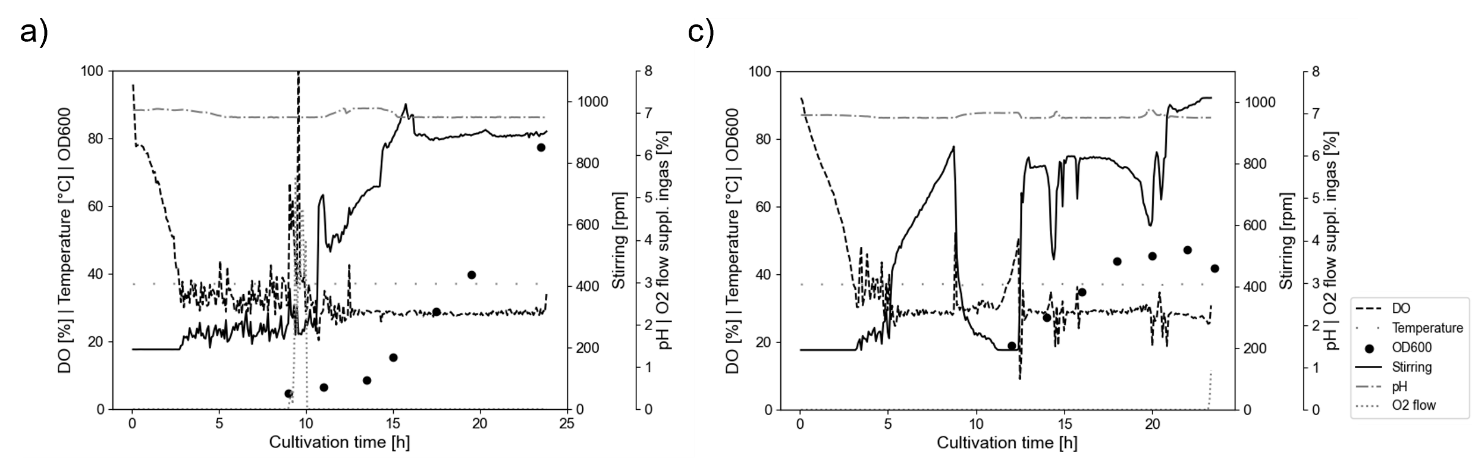


Figure S 1 (a) Inline and offline data recorded during the expression of FGG as well as (b) Inline and offline data during the cultivation of the empty vector control.

## ^1^H-NMR spectra


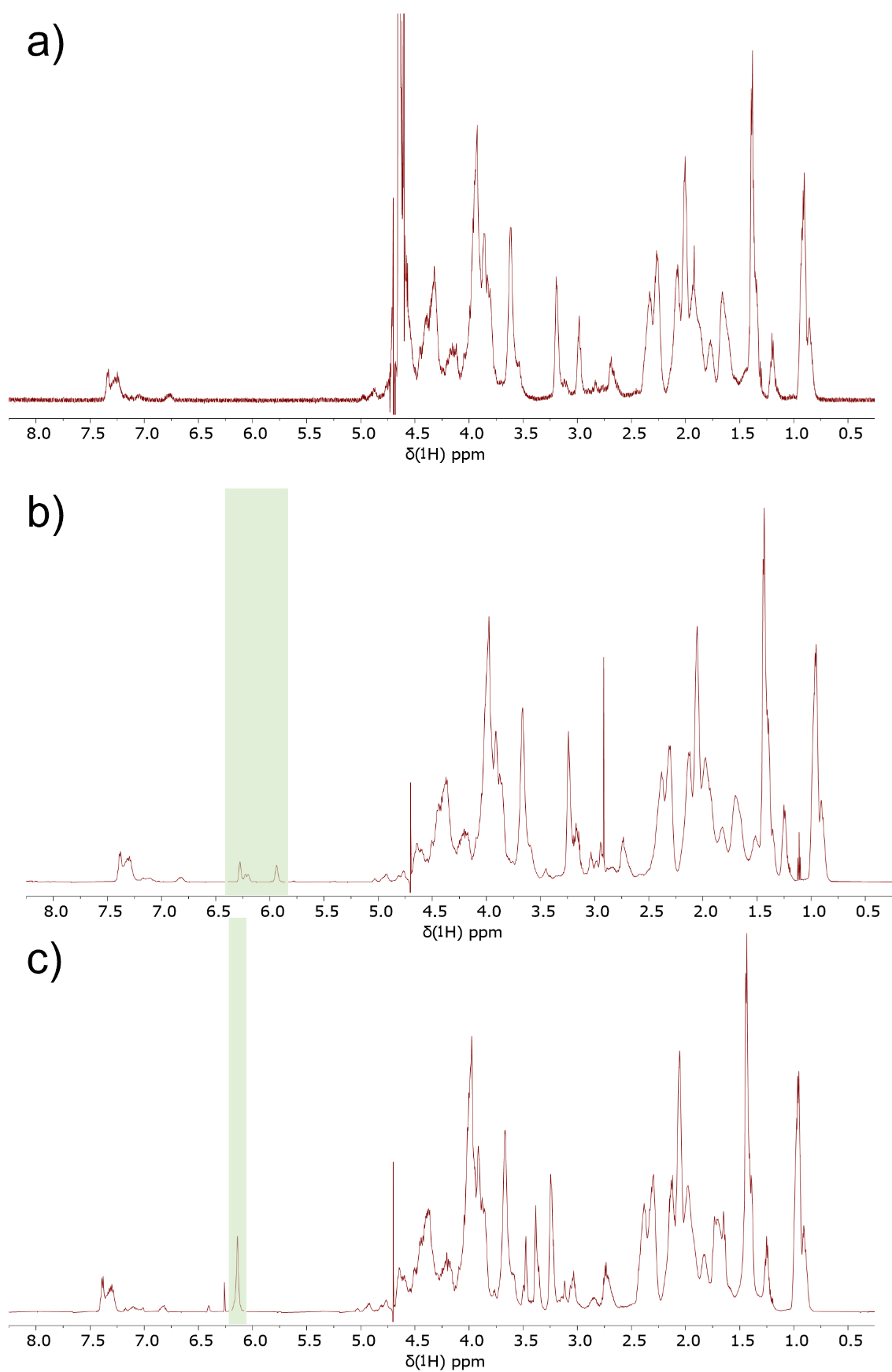


Figure S 2 ^1^H-NMR spectra of Gelatin (a), and GelNB synthesized by EDC-NHS coupling (b) and carbic anhydride-based synthesis (c). Green Boxes show peaks associated with norbornene functionalization absent in Gelatin used for identification of successful functionalization.

Table S1 Reagent amounts added per gram gelatin and resulting DoF for the different functionalization routes for GelNB.

| Batch | NB carboxylic acid  [g g^-1^] | EDC  [g g^-1^] | NHS  [g g^-1^] | Carbic anhydride [mg g^-1^] | DoF |
| --- | --- | --- | --- | --- | --- |
| GelNB (EDC/NHS) | 0.0736 | 0.204 | 0.062 | - | 76 % |
| GelNB (anhydride) | - | - | - | 130 | 68 % |


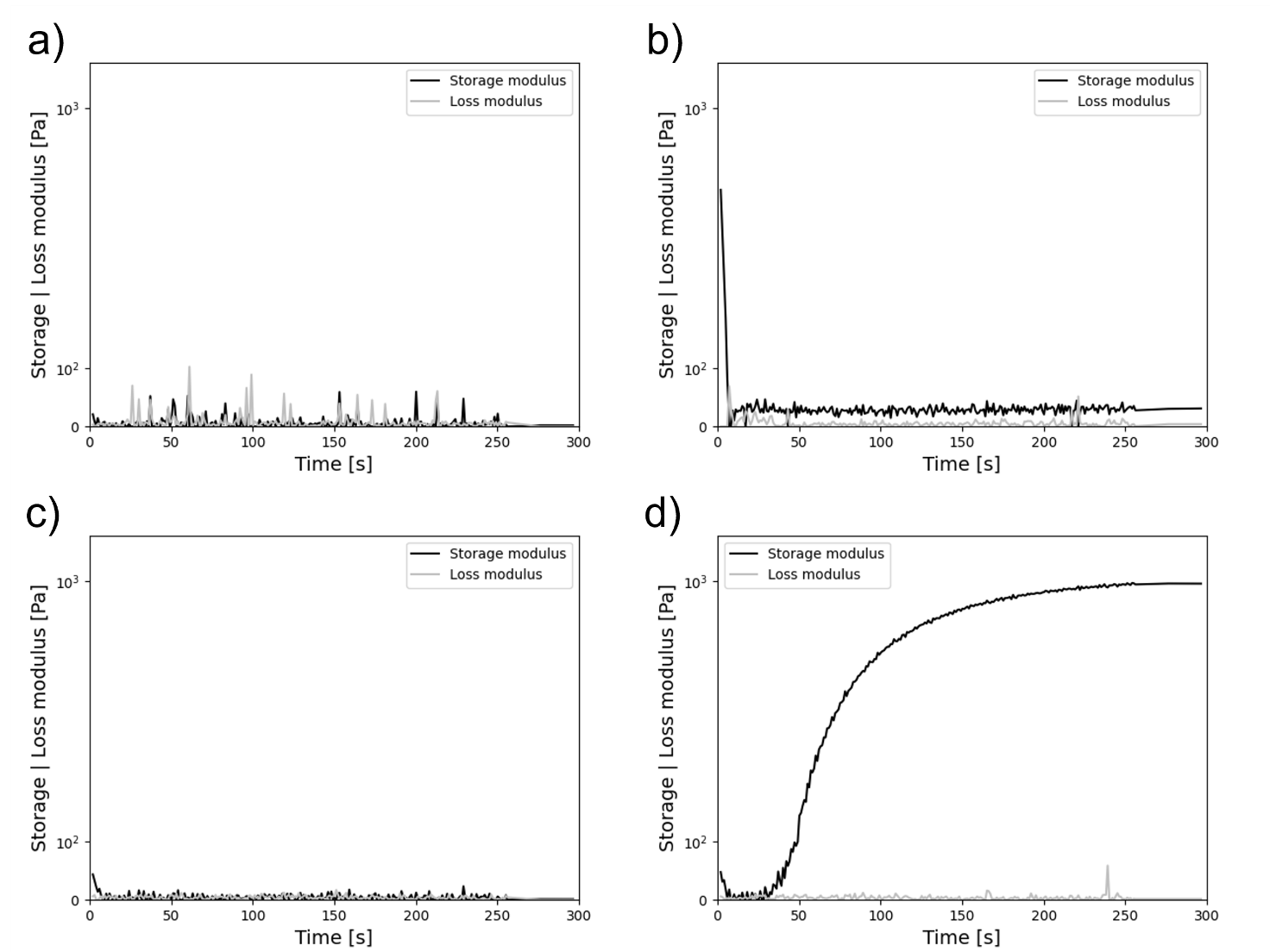


Figure S 3 Storage and loss modulus of (a) Empty vector control (b) FGG, (c) FGG with gelatin (5% (w/v)) (d) FGG with GelNB (5% (w/v)) during in situ polymerization by UV light exposure in presence of 0.1% LAP.


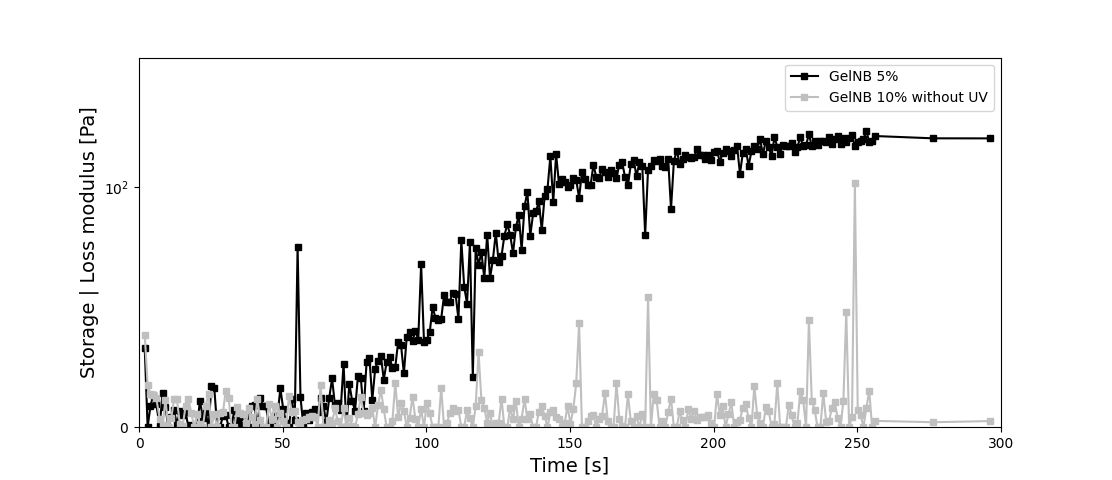


Figure S 4 Homopolymerization of GelNB (5%) in presence of 0.1% LAP during UV irradiation compared to GelNB (10%) in presence of 0.1% LAP without UV exposure.


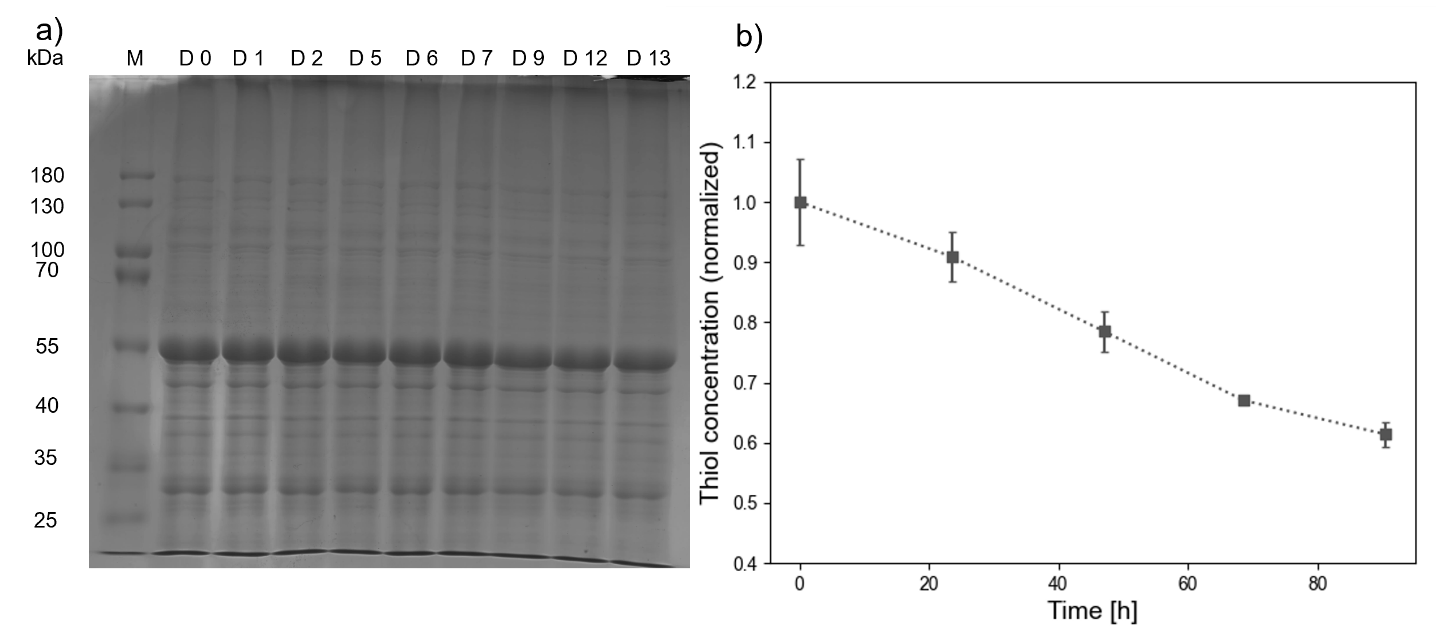


Figure S 5 Stability analysis of FGG in solubilization buffer. (a) SDS-PAGE of the FGG protein stored in solubilization buffer at 4 °C for 1-13 days (D 1 to D 13) with the protein directly after solubilization (D 0) as reference. (b) thiol groups detected by Ellman’s assay over extended storage time in solubilization buffer normalized to the average of thiol groups detected in the sample directly after solubilization (boxes show averages measured, the line does not reflect actual data points but rather provides guidance of the general trend) n=3.
